# Ag_2_WO_4_ nanorods decorated with AgI nanoparticles: Novel and efficient visible-light-driven photocatalysts for the degradation of water pollutants

**DOI:** 10.3762/bjnano.9.123

**Published:** 2018-04-27

**Authors:** Shijie Li, Shiwei Hu, Wei Jiang, Yanping Liu, Yu Liu, Yingtang Zhou, Liuye Mo, Jianshe Liu

**Affiliations:** 1 Key Laboratory of Health Risk Factors for Seafood of Zhejiang Province, Institute of Innovation & Application, Zhejiang Ocean University, Zhoushan, Zhejiang Province, 316022, China; 2 Department of Environmental Engineering, Zhejiang Ocean University, Zhoushan, Zhejiang Province, 316022, China,; 3 State Environmental Protection Engineering Center for Pollution Treatment and Control in Textile Industry, College of Environmental Science and Engineering, Donghua University, Shanghai 201620, China

**Keywords:** AgI/Ag_2_WO_4_, nanocomposites, photocatalysis, visible light

## Abstract

To develop efficient and stable visible-light-driven (VLD) photocatalysts for pollutant degradation, we synthesized novel heterojunction photocatalysts comprised of AgI nanoparticle-decorated Ag_2_WO_4_ nanorods via a facile method. Various characterization techniques, including XRD, SEM, TEM, EDX, and UV–vis DRS were used to investigate the morphology and optical properties of the as-prepared AgI/Ag_2_WO_4_ catalyst. With AgI acting as the cocatalyst, the resulting AgI/Ag_2_WO_4_ heterostructure shows excellent performance in degrading toxic, stable pollutants such as rhodamine B (RhB), methyl orange (MO) and *para*-chlorophenol (4-CP). The high performance is attributed to the enhanced visible-light absorption properties and the promoted separation efficiency of charge carriers through the formation of the heterojunction between AgI and Ag_2_WO_4_. Additionally, AgI/Ag_2_WO_4_ exhibits durable stability. The active species trapping experiment reveals that active species (O_2_^•−^ and h^+^) dominantly contribute to RhB degradation. The AgI/Ag_2_WO_4_ heterojunction photocatalyst characterized in this work holds great potential for remedying environmental issues due to its simple preparation method and excellent photocatalytic performance.

## Introduction

The development of high-performance novel photocatalysts for the degradation of pollutants has received great interest due to the worsening of environmental pollution [1–11]. However, achieving high efficiency for photocatalytic conversion under natural sunlight irradiation is still a great challenge because many catalysts only respond to ultraviolet (UV) light [5,12]. Exploring photocatalysts that can be driven by visible light, which comprises 43% of solar energy, is important for practical application [13–20].

Silver-containing compounds such as AgI, Ag_2_CrO_4_, Ag_2_O, Ag_3_PO_4_ etc., have proven to be efficient VLD photocatalysts [21–35]. Among these photocatalysts, Ag_2_WO_4_ presents good photocatalytic performance for dye degradation under light irradiation [30–31,36–37]. Unfortunately, due to its wide bandgap of about 3.1 eV, Ag_2_WO_4_ has limited photocatalytic activity under sunlight, which severely limits its application and illustrates the urgency for optimization of Ag_2_WO_4_ to overcome these disadvantages [38–42].

The integration of VLD components with wide bandgap semiconductors having well-matched energy bands has provided a new opportunity for the development of VLD photocatalysts [12]. As a consequence, some Ag_2_WO_4_-based composites containing VLD components such as Ag_2_S/Ag_2_WO_4_ [40], C_3_N_4_/Ag_2_WO_4_ [39], Bi_2_MoO_6_/Ag_2_WO_4_/Ag [42] etc., have been reported to show improved VLD performance in the degradation of pollutants. To the best of our knowledge, application of AgI/Ag_2_WO_4_ as a VLD photocatalyst for the degradation of toxic pollutants remains unreported.

In this study, to enhance the photocatalytic performance of Ag_2_WO_4_, AgI (possessing matched energy band levels) was chosen as a suitable component to combine with Ag_2_WO_4_, AgI/Ag_2_WO_4_ heterojunctions at different mole ratios. These heterojunctions were prepared via an in situ ion-exchange approach, utilizing Ag_2_WO_4_ nanorods as the Ag source. The as-prepared AgI/Ag_2_WO_4_ heterojunctions exhibited remarkably higher photocatalytic activity than pure Ag_2_WO_4_ toward the degradation of rhodamine B (RhB), methyl orange (MO) and *para*-chlorophenol (4-CP) under visible light. Based on a systematic characterization and study, a possible photocatalytic mechanism over AgI/Ag_2_WO_4_ was also elucidated in this work.

## Results and Discussion

### Preparation and characterization of catalysts

Ag_2_WO_4_ nanorods decorated with AgI nanoparticles were prepared via an in situ anion-exchange method. Ag_2_WO_4_ nanorods were first synthesized by mixing AgNO_3_ and Na_2_WO_4_ aqueous solutions at room temperature [37]. Subsequently, AgI nanoparticles were readily anchored onto Ag_2_WO_4_ nanorods via an in situ anion-exchange between I^−^ in the solution and the lattice W_2_O_4_^2−^ in Ag_2_WO_4_. The resulting catalysts were denoted as 0.1AgI/Ag_2_WO_4_, 0.2AgI/Ag_2_WO_4_, 0.3AgI/Ag_2_WO_4_, and 0.4AgI/Ag_2_WO_4_, respectively.

**Figure 1:**
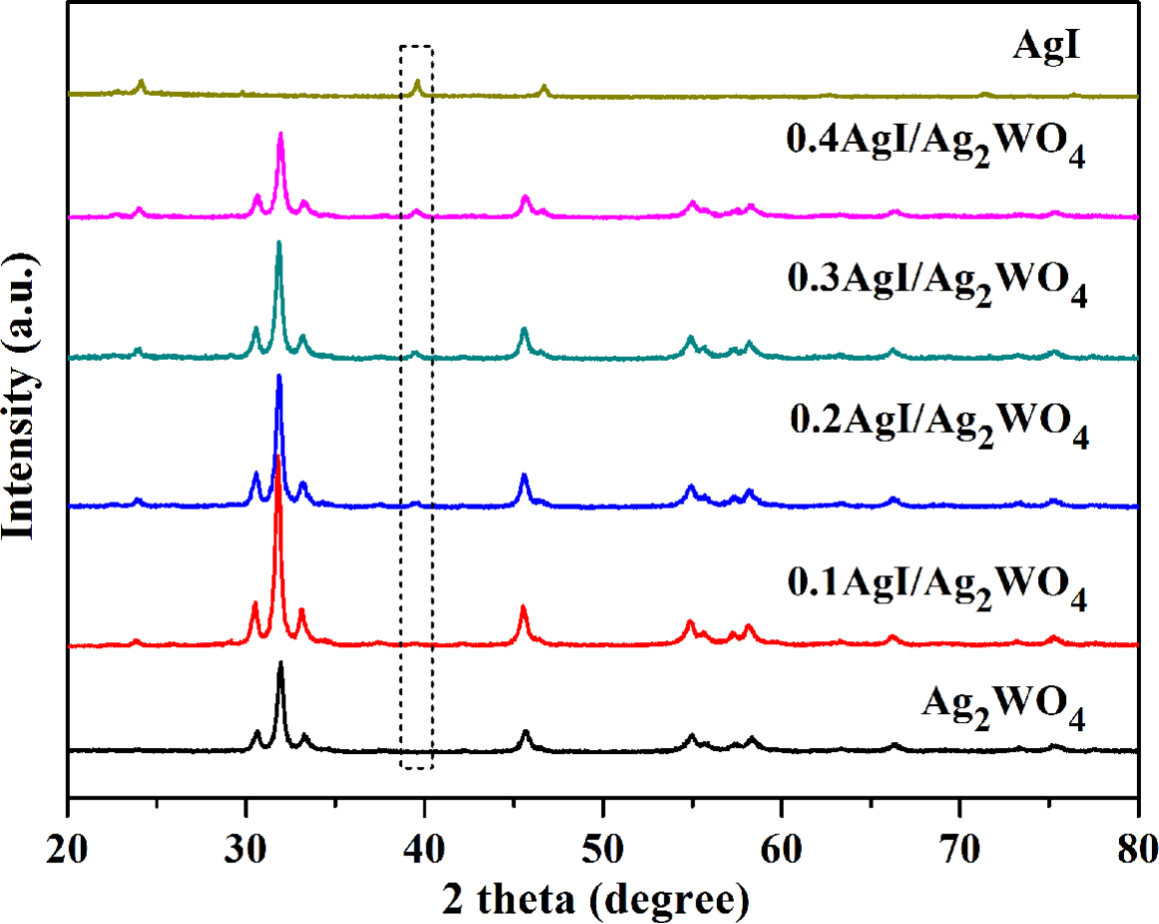
XRD patterns of Ag_2_WO_4_, AgI, 0.1AgI/Ag_2_WO_4_, 0.2AgI/Ag_2_WO_4_, 0.3AgI/Ag_2_WO_4_, and 0.4AgI/Ag_2_WO_4_.

The XRD patterns of pure Ag_2_WO_4_, pure AgI, and AgI/Ag_2_WO_4_ heterojunctions are displayed in Figure 1. Pure Ag_2_WO_4_ is in its orthorhombic structure (JCPDS no. 70-1719) and exhibits several strong peaks at 30.3°, 31.8°, 33.1, 45.6°, 54.8° and 58.3°, which can be indexed to (002), (231), (400), (402), (361) and (333) diffraction planes, respectively [31,41]. The diffraction peaks of pure AgI match well with those of the standard hexagonal phase (JCPS no 29-1154) [43]. It can be seen that all the AgI/Ag_2_WO_4_ composites display both Ag_2_WO_4_ and AgI phases. Of note is that the diffraction peak intensity of AgI becomes stronger with increasing AgI content, confirming the formation of AgI/Ag_2_WO_4_ heterojunctions.

**Figure 2:**
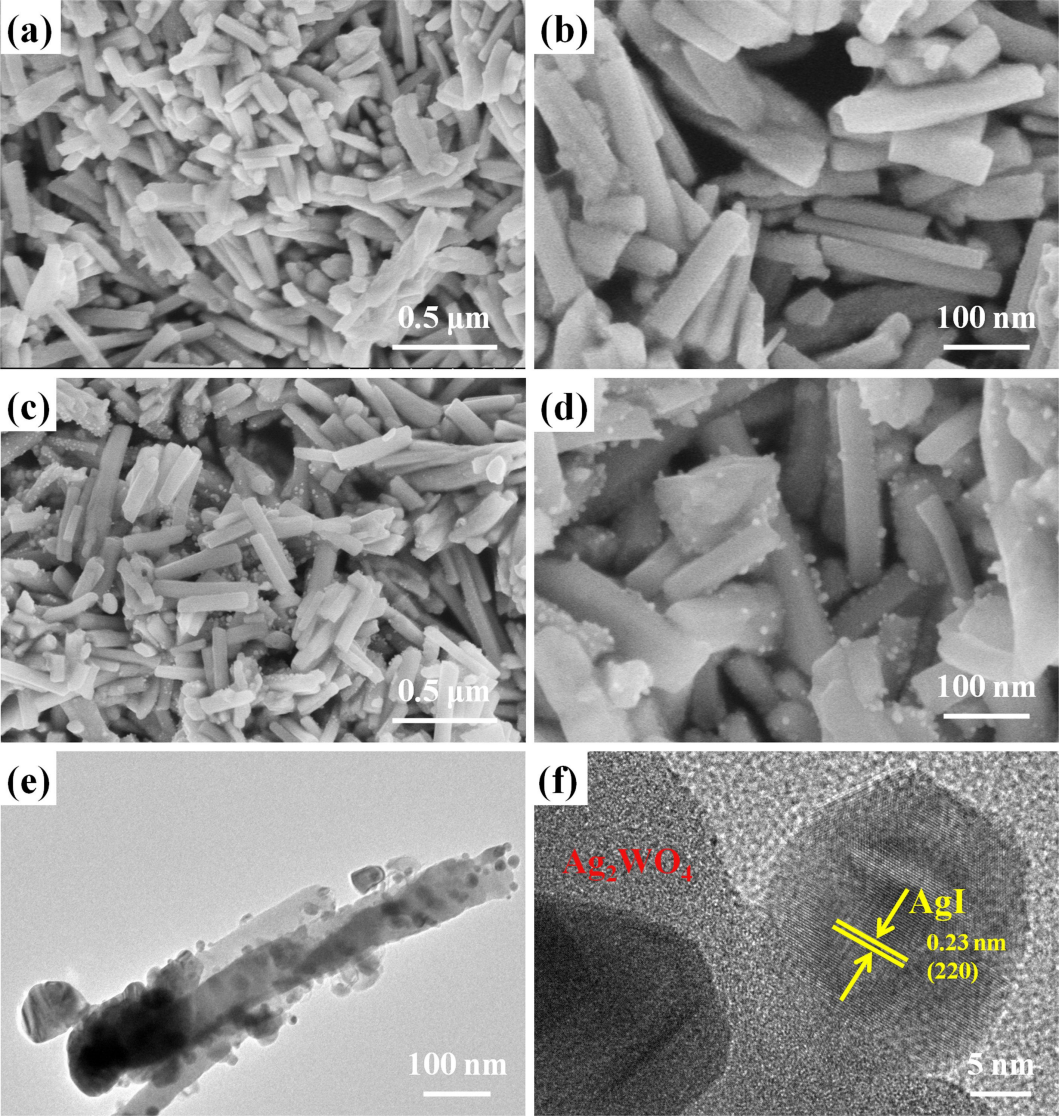
SEM (a, b) images of Ag_2_WO_4_; SEM (c, d), TEM (e), and HRTEM (f) images of 0.3AgI/Ag_2_WO_4_.

Scanning electron microscopy (SEM) and transmission electron microscopy (TEM) were used to investigate the morphology and microstructure of AgI/Ag_2_WO_4_ heterojunctions (Figure 2). Figure 2a–d presents the SEM images of Ag_2_WO_4_ (Figure 2a,b) and the representative 0.3AgI/Ag_2_WO_4_ (Figure 2c,d). It can be seen that bare Ag_2_WO_4_ consists of nanorods (length: 0.3–0.8 μm) with smooth surfaces (Figure 2a,b). After in situ anion-exchange between I^-^ and the lattice W_2_O_4_^2−^, small AgI nanoparticles (diameter: 20–40 nm) are uniformly coated on the surface of Ag_2_WO_4_ nanorods, signifying the formation of the AgI/Ag_2_WO_4_ core–shell heterostructure. To more clearly observe the microstructure of the AgI/Ag_2_WO_4_ composite, the TEM and high-resolution TEM (HRTEM) images are shown in Figure 2e,f. It can be seen that many nanoparticles are deposited on the surface of the Ag_2_WO_4_ nanorods (Figure 2e). The HRTEM image (Figure 2f) shows that one set of lattice fringes can be observed. The lattice fringe of 0.23 nm matches well with the (220) plane of AgI. No lattice fringe correlated to Ag_2_WO_4_ can be distinguished, which is in accordance with the previous reports [40].

**Figure 3:**
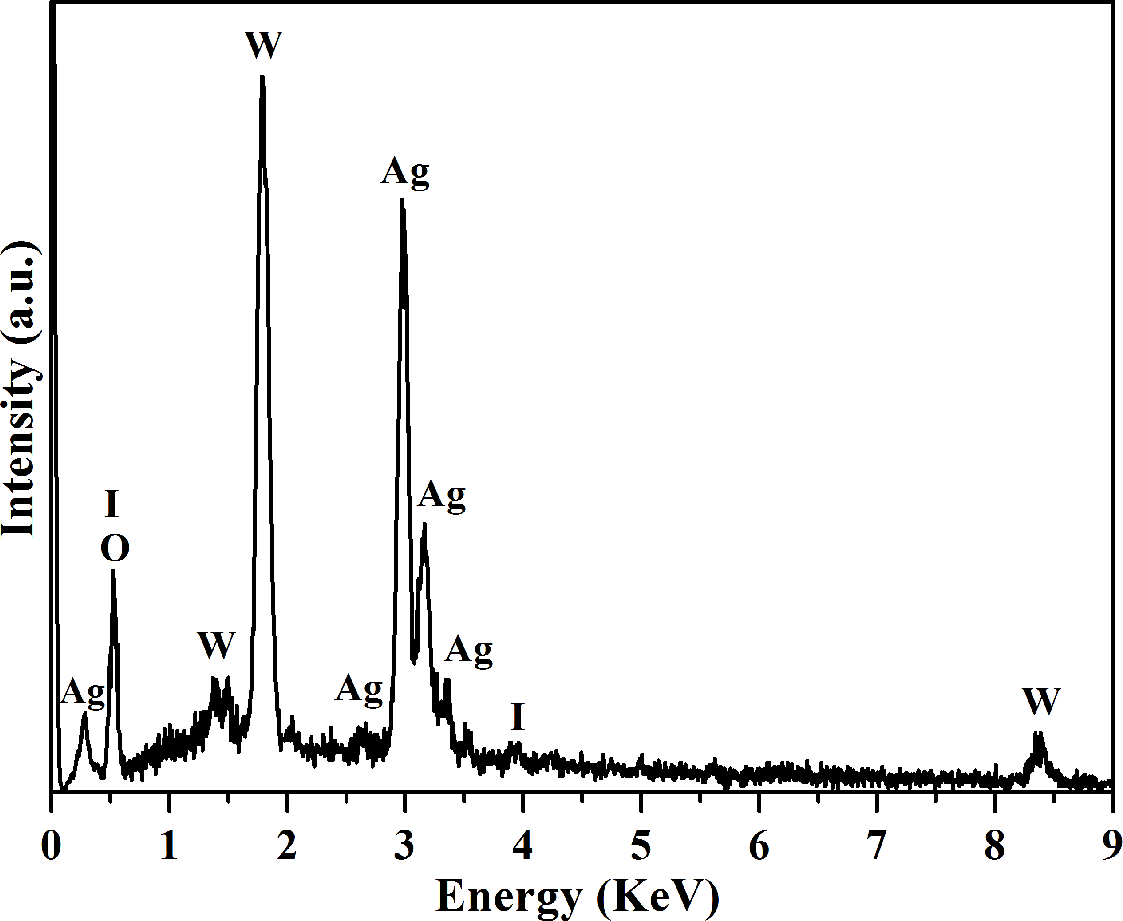
Energy-dispersive X-ray (EDX) spectrum of 0.3AgI/Ag_2_WO_4_.

In addition, the composition of 0.3AgI/Ag_2_WO_4_ was further identified by energy-dispersive X-ray spectroscopy (EDX). As shown in Figure 3, 0.3AgI/Ag_2_WO_4_ is composed of Ag, I, W and O elements. The results further confirm the formation of the AgI/Ag_2_WO_4_ heterojunctions, thereby facilitating the charge transfer between them [12].

**Figure 4:**
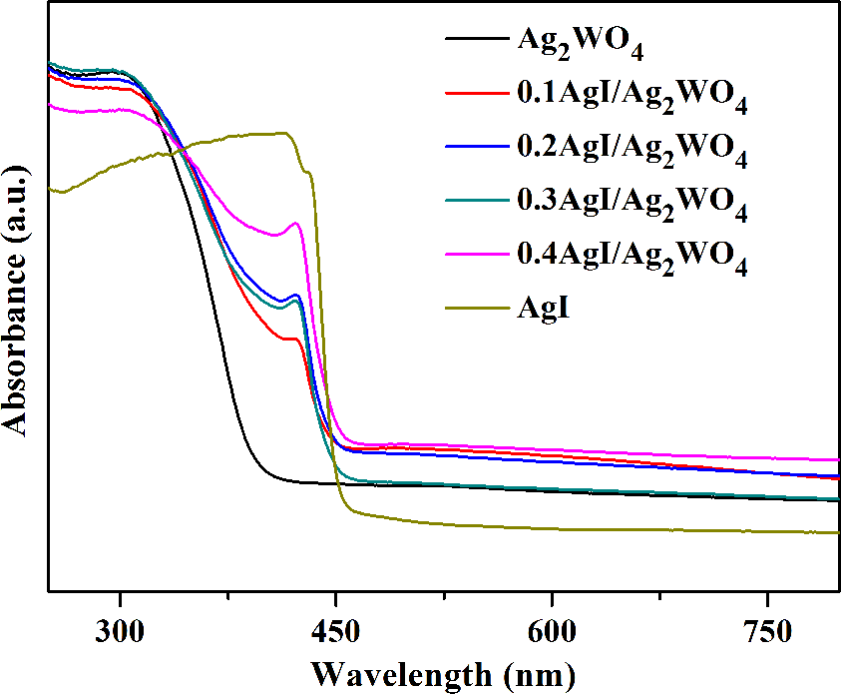
UV–vis diffuse reflectance spectra of Ag_2_WO_4_, AgI, 0.1AgI/Ag_2_WO_4_, 0.2AgI/Ag_2_WO_4_, 0.3AgI/Ag_2_WO_4_, and 0.4AgI/Ag_2_WO_4_.

Subsequently, the optical properties of as-prepared photocatalysts were investigated by UV–vis diffuse reflectance spectroscopy (DRS) analysis (Figure 4). As indicated from Figure 4, pure Ag_2_WO_4_ has a clear absorption edge at about 400 nm [31], while AgI has a broader absorption band with the absorption edge located at around 460 nm [22]. For AgI/Ag_2_WO_4_ heterojunctions, a pronounced enhancement in visible-light absorption range is achieved when AgI content was progressively increased. This suggests that the introduction of AgI nanoparticles can optimize the light absorption capacity owing to the formation of a nanojunction between AgI and Ag_2_WO_4_. These facts indicate that AgI/Ag_2_WO_4_ heterojunctions can harvest more light and thus can be expected to be efficient VLD photocatalysts.

### Photocatalytic performance

**Figure 5:**
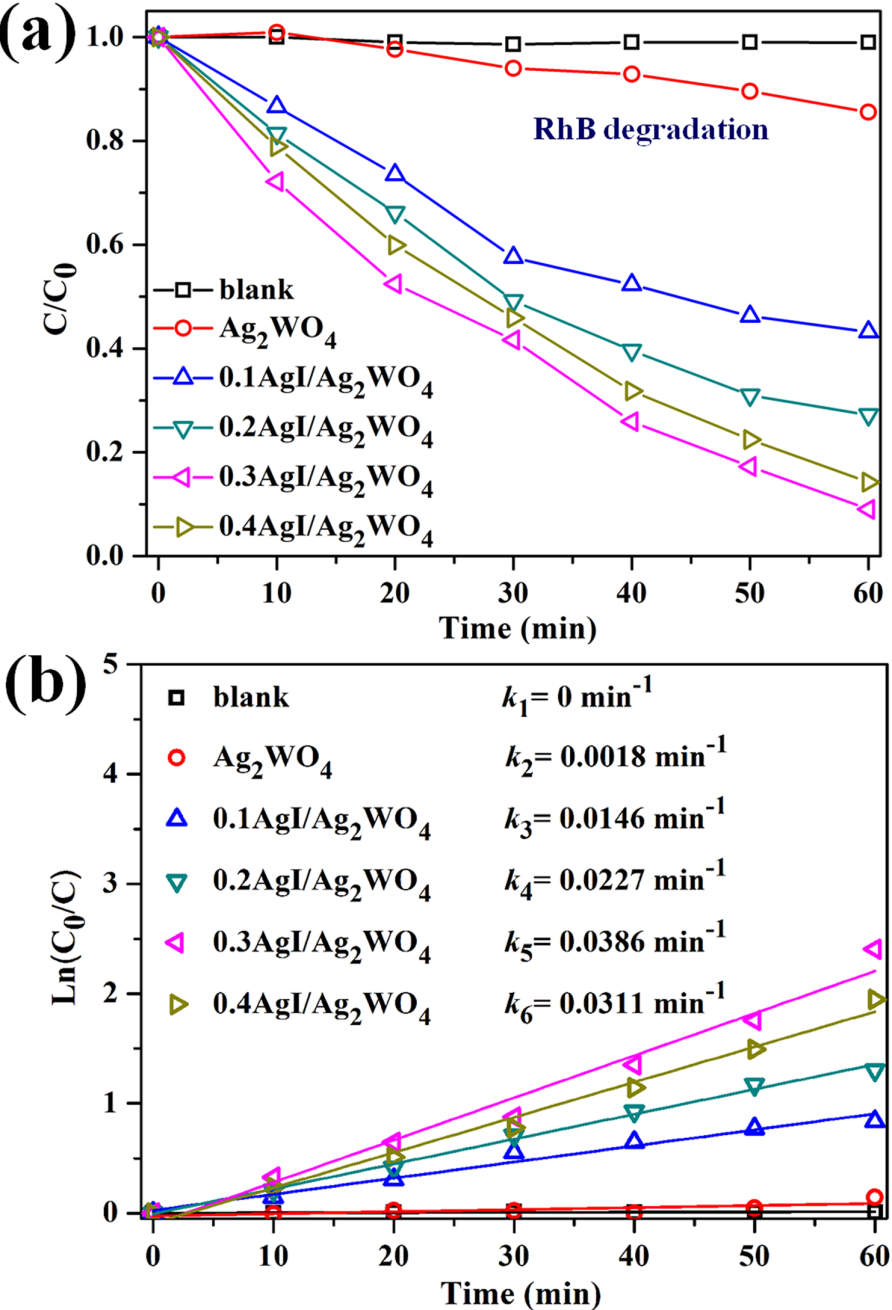
(a) The photocatalytic degradation and (b) degradation rate constants of RhB using different catalysts (10 mg) under visible light.

RhB, MO, and 4-CP, three types of toxic pollutants with stable chemical structures, were used to evaluate the activity of AgI/Ag_2_WO_4_ heterojunctions. Figure 5a shows the visible-light degradation results of RhB (10 mg L**^−^**^1^) over as-prepared samples. Bare Ag_2_WO_4_ exhibits poor visible-light photocatalytic activity with an RhB degradation rate of 16.2% after 60 min irradiation due to the unsatisfactory visible-light absorption and fast recombination of photoinduced charge carriers [31,38,44–45]. After hybridization of AgI, all AgI/Ag_2_WO_4_ heterojunctions (0.1AgI/Ag_2_WO_4_, 0.2AgI/Ag_2_WO_4_, 0.3AgI/Ag_2_WO_4_, and 0.4AgI/Ag_2_WO_4_) show greatly improved photocatalytic activity compared with pure Ag_2_WO_4_, and their degradation efficiency (within 60 min of reaction) are 56.8%, 72.7%, 91.3% and 85.6%, respectively. Among these composites, 0.3AgI/Ag_2_WO_4_ shows the highest performance. Apparently, the introduction of a proper amount of AgI can markedly facilitate the separation of electron–hole pairs, leading to the marked photocatalytic activity, which is in accordance with previous reports. As the amount of AgI is further increased, the activity of 0.4AgI/Ag_2_WO_4_ decreases, indicating that excessive AgI is unfavorable for photocatalytic reaction. The possible reason is that a large amount of AgI particles having a larger diameter could interfere with the light absorption of reactive sites. It has also been found that the degradation process of RhB can be fitted well with the apparent first-order model (Figure 5b). As we can see, the *k* value for RhB decomposition over 0.3AgI/Ag_2_WO_4_ is about 0.0386 min**^−^**^1^, which is much higher than those over other samples.

**Figure 6:**
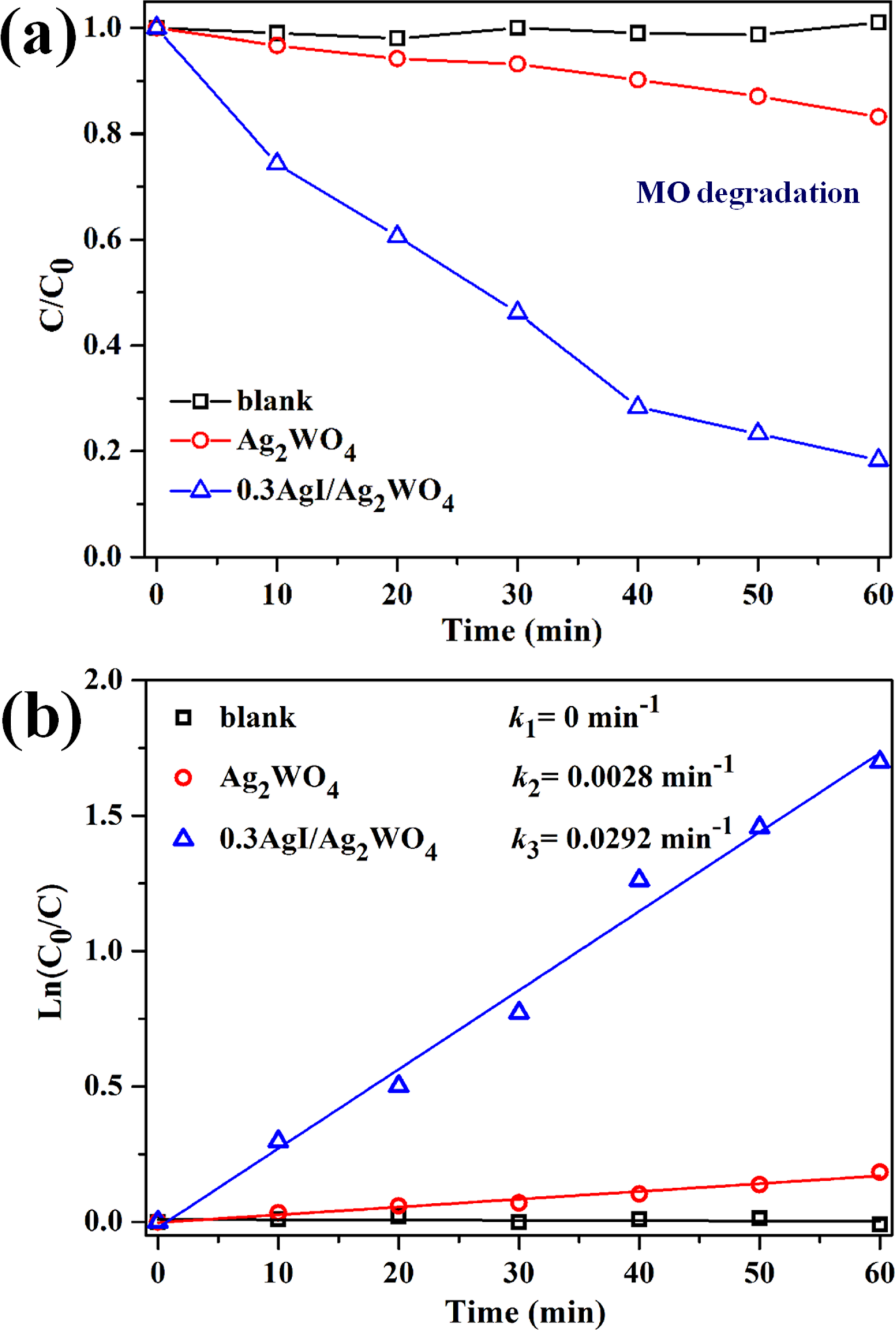
(a) The photocatalytic degradation and (b) degradation rate constants of MO (100 mL, 5 mg L^−1^) using the catalysts (10 mg).

Besides RhB, MO (Figure 6a) and 4-CP (Figure S1, Supporting Information File 1) also could be efficiently degraded by 0.3AgI/Ag_2_WO_4_ under visible light, indicating the outstanding photocatalytic activity of 0.3AgI/Ag_2_WO_4_. In addition, the degradation rate constant of MO (Figure 6b) and 4-CP (Figure S1b, Supporting Information File 1) over catalysts were also calculated by the pseudo-first-order model. It is found that 0.3AgI/Ag_2_WO_4_ still achieves the highest apparent rate constant (0.0292 min^−1^ for MO degradation and 0.0129 min^−1^ for 4-CP degradation) among all these samples.

**Figure 7:**
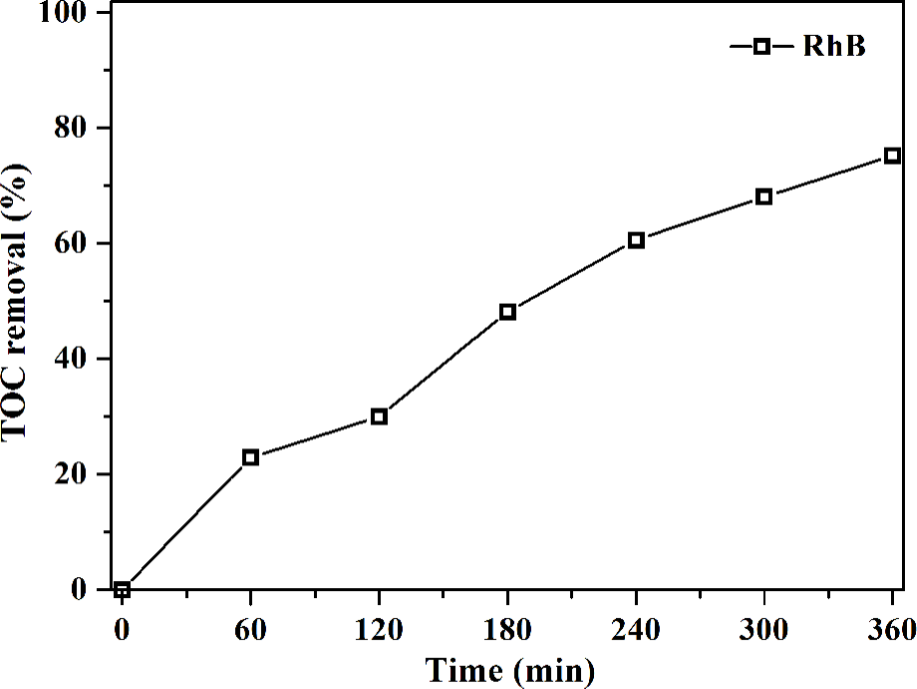
Total organic carbon (TOC) removal during the photocatalytic degradation of RhB in the presence of 0.3AgI/Ag_2_WO_4_.

The mineralization of organic pollutants is crucial for pollutant treatment [46]. Thus, the total organic carbon (TOC) removal efficiency of RhB over 0.3AgI/Ag_2_WO_4_ was examined (Figure 7). After 360 min of reaction, the TOC removal efficiency reached 67.2%, signifying that 0.3AgI/Ag_2_WO_4_ can effectively mineralize RhB.

**Figure 8:**
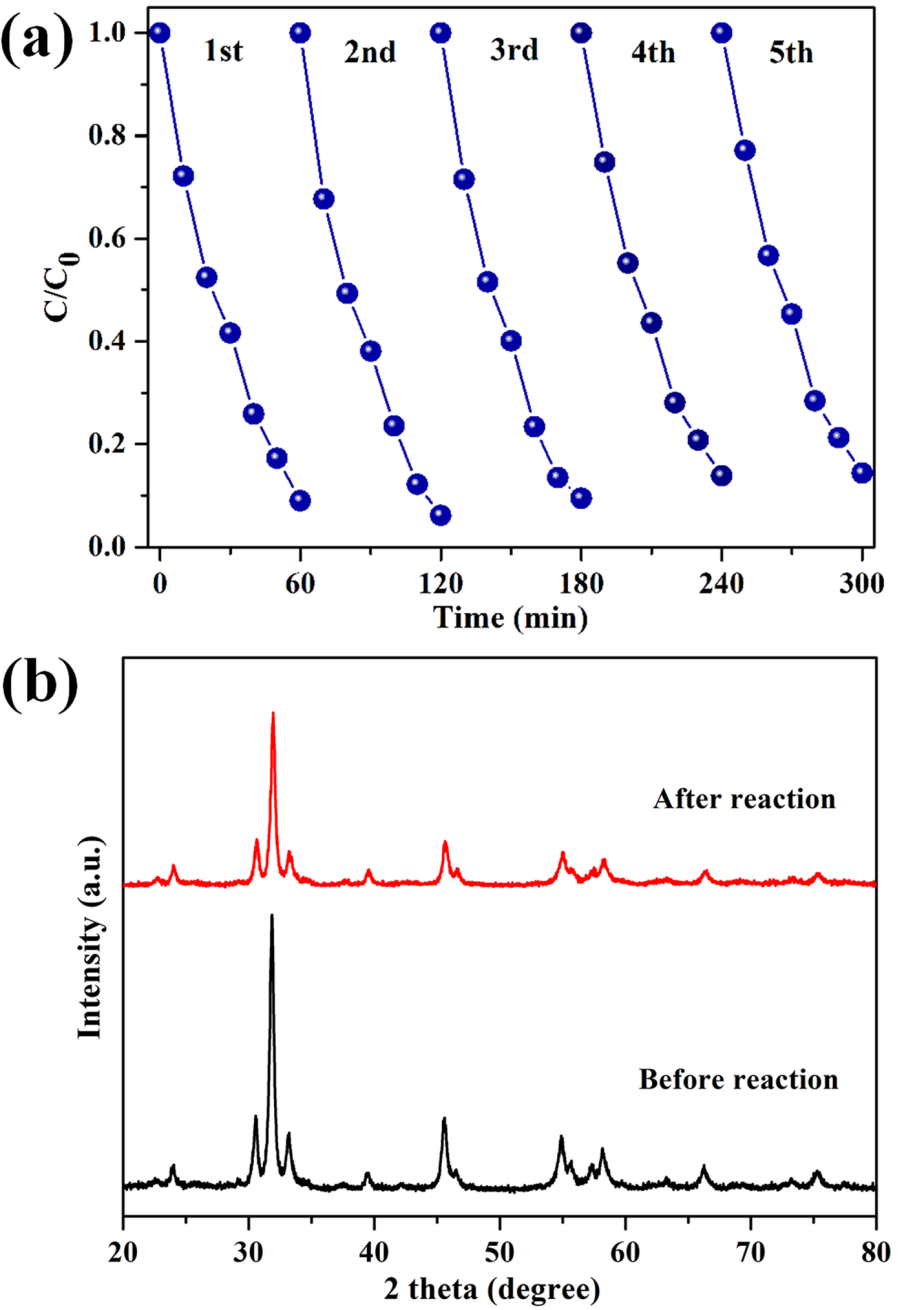
(a) The cycled photocatalytic degradation of RhB over 0.3AgI/Ag_2_WO_4_; (b) XRD patterns of the fresh and used 0.3AgI/Ag_2_WO_4_.

The operational lifetime of the photocatalysts is crucial for practical application [47]. To reveal the durability of 0.3AgI/Ag_2_WO_4_, the cycling photocatalytic degradation of RhB was performed. As shown in Figure 8a, no apparent activity decrease was observed after five successive runs, demonstrating the good stability of the catalyst. Furthermore, the XRD pattern of the used 0.3AgI/Ag_2_WO_4_ is similar to that of the fresh one (Figure 8b). These facts suggest that 0.3AgI/Ag_2_WO_4_ possesses long-term stability for photocatalytic reaction.

### Photocatalytic mechanism

**Figure 9:**
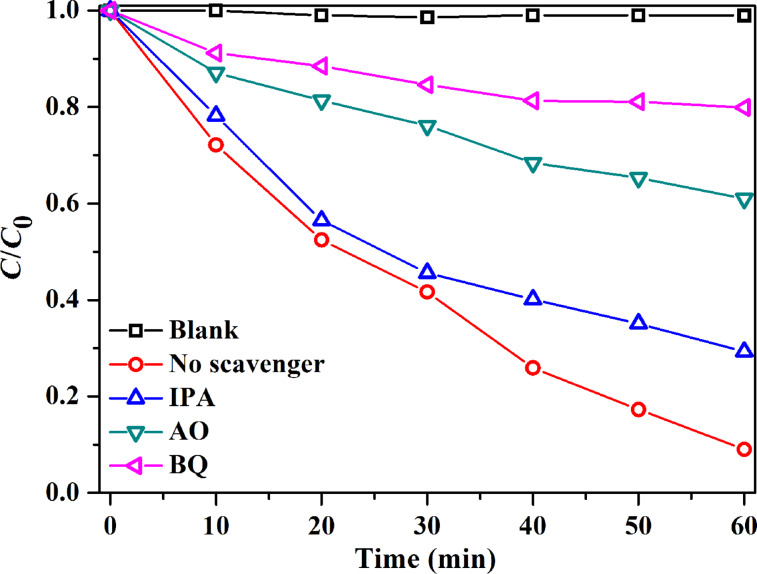
Active-species trapping tests over 0.3AgI/Ag_2_WO_4_.

To elucidate the degradation mechanism, active-species trapping tests were performed during RhB degradation over 0.3AgI/Ag_2_WO_4_ (Figure 9) [13,48]. Figure 9 shows the effects of various trapping agents on the RhB degradation efficiency under visible-light irradiation. When IPA was introduced, the RhB degradation efficiency slightly reduced from 91.3 % to 70.7%, suggesting that very little •OH was involved in the reaction. However, when benzoquinone (a superoxide radical (•O_2_^−^) scavenger) or ammonium oxalate (a hole radical (h^+^) scavenger) was introduced, the degradation rate of RhB was severely depressed. That is, •O^2−^, •OH, and h^+^ were generated in the 0.3AgI/Ag_2_WO_4_ mediated degradation system, but •O^2−^ and h^+^ played a more crucial role in RhB degradation.

**Figure 10:**
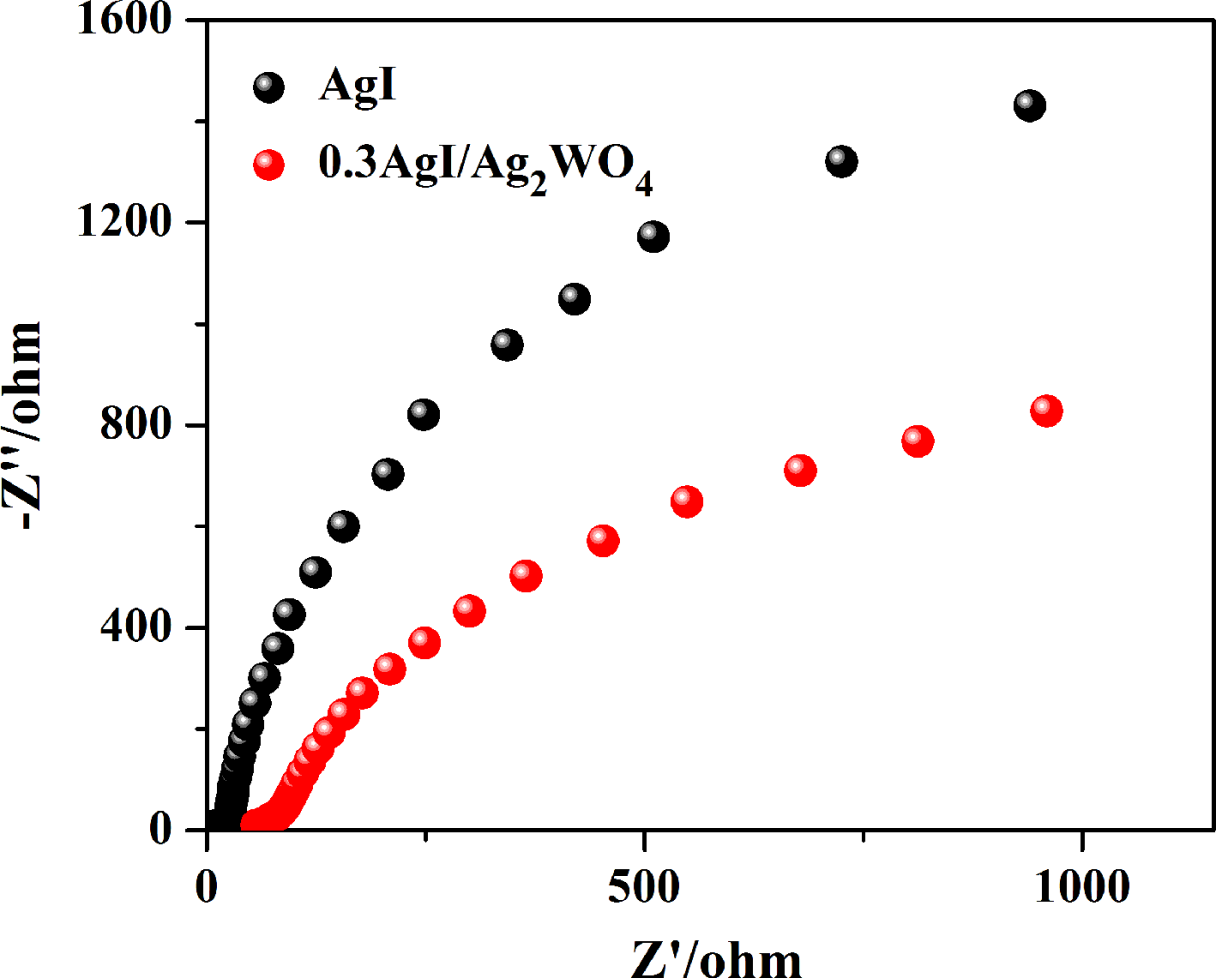
Electrochemical impedance spectroscopy (EIS) Nyquist plots of AgI and 0.3AgI/Ag_2_WO_4_.

Electrochemical impedance spectroscopy (EIS) measurement was applied to study the charge transport and separation [49]. A smaller arc radius commonly signifies a higher charge transport rate. As displayed in Figure 10, the arc radius of 0.3AgI/Ag_2_WO_4_ is smaller than that of AgI, suggesting that 0.3AgI/Ag_2_WO_4_ holds a higher charge transfer rate and a more effective separation of charge carriers.

**Figure 11:**
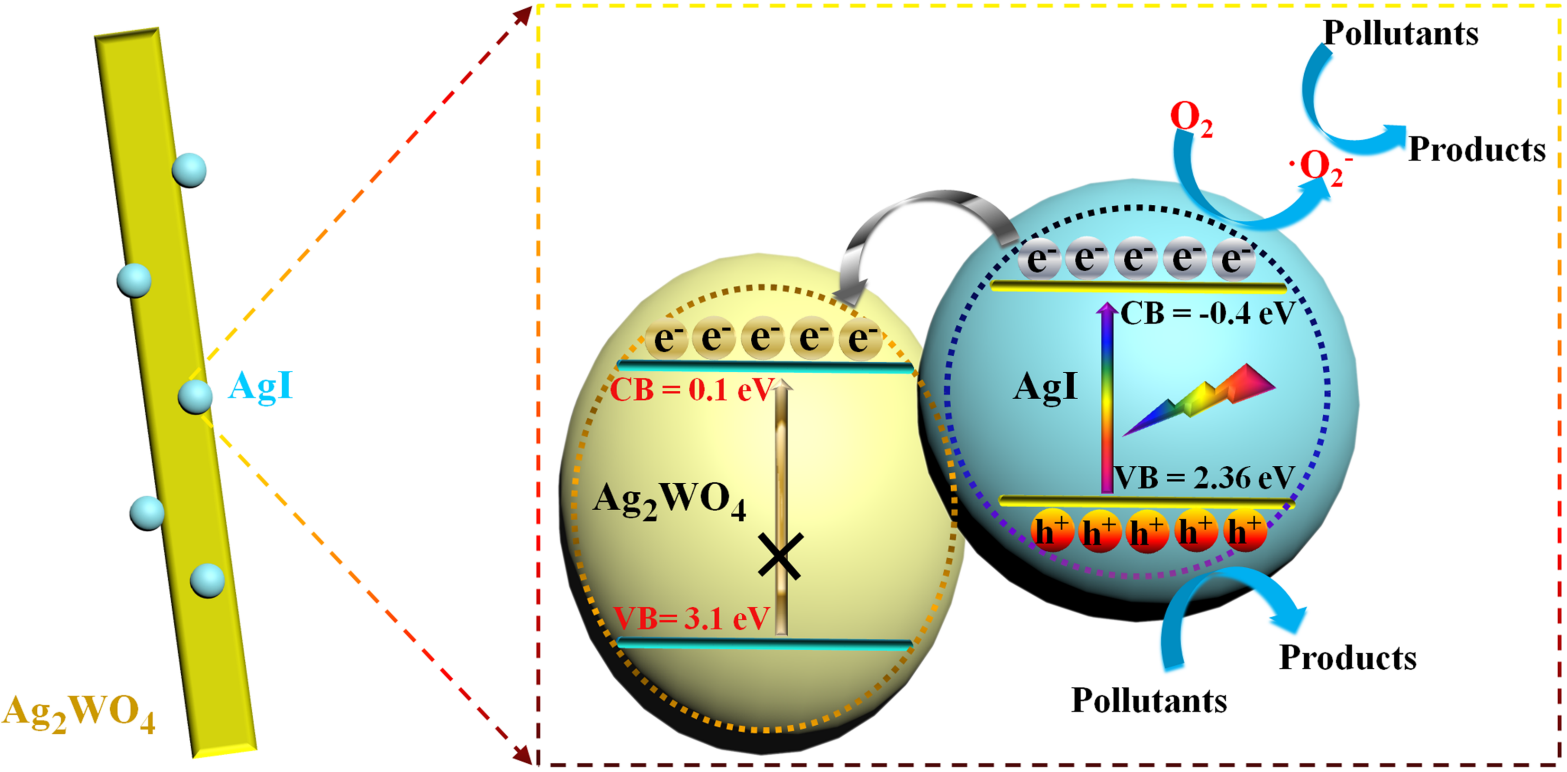
Schematic diagram of electron–hole pair separation and the possible reaction mechanism over the AgI/Ag_2_WO_4_ heterojunction under visible-light irradiation.

On the basis of the above discussion, the excellent photocatalytic activity of AgI/Ag_2_WO_4_ is concluded to be due to the broadening of the photo-absorption range from the ultraviolet to the visible light range (Figure 4) and the formation of a heterojunction between AgI and Ag_2_WO_4_. The matched band alignments lead to a fascinating separation and transfer of the photo-generated electrons and holes (Figure 11) [50–52]. A heterostructure is well constructed after the in situ growth of AgI on Ag_2_WO_4_. Under visible light irradiation, AgI is excited to produce electrons and holes. Given the negative potential of conduction band (CB) of AgI to that of Ag_2_WO_4_, electrons tend to migrate from the CB of AgI to that of Ag_2_WO_4_, whereby the separation rate of electron–hole pairs is boosted. Consequently, the accumulated electrons in the CB of AgI (and more holes left behind in the valence band (VB)) could readily attack the pollutant molecules, resulting in the remarkable photocatalytic performance of AgI/Ag_2_WO_4_.

## Conclusion

In summary, a novel heterojunction photocatalyst comprised of AgI nanoparticle-decorated Ag_2_WO_4_ nanorods exhibiting remarkable photocatalytic performance has been prepared via a facile method. This resulting AgI/Ag_2_WO_4_ catalyst exhibits exceptionally high and stable photocatalytic activity for the degradation of RhB, MO and 4-CP due to its extended light absorption range and the formation of a heterojunction between AgI and Ag_2_WO_4_. This work not only offers a high-efficiency AgI/Ag_2_WO_4_ heterojunction photocatalyst, but also provides new inspiration for the development of visible-light-driven Ag-based heterojunction photocatalysts.

## Experimental

### Photocatalyst synthesis

All reagents were purchased from Shanghai Sinopharm Chemical Reagent Ltd. and used as received.

Ag_2_WO_4_ nanorods were prepared according to a previous report [41]. Briefly, AgNO_3_ (0.01 mol L^−1^, 100 mL) and Na_2_WO_4_ (0.005 mol L^−1^, 100 mL) aqueous solutions were first prepared. Then, the AgNO_3_ aqueous solution was slowly poured into Na_2_WO_4_ aqueous solution and incubated at room temperature for 12 h in the dark. Finally, the white precipitate was collected, washed successively with distilled water, and dried in vacuum at 60 °C for 12 h.

The AgI/Ag_2_WO_4_ nanorods were prepared by an in situ anion-exchange reaction of Ag_2_WO_4_ nanorods in KI aqueous solution at a temperature of 60 °C in the dark. In a typical reaction, KI (0.1 mmol, 0.2 mmol, 0.3 mmol, 0.4 mmol) was dissolved separately in 60 mL distilled water and stirred to obtain a clear solution. Then, the as-prepared Ag_2_WO_4_ nanorods (1 mmol) were dispersed respectively in each of the above solutions at 60 °C under stirring for 5 h. After reaction, the products with different amounts of KI (0.1 mmol, 0.2 mmol, 0.3 mmol, 0.4 mmol) were collected, washed, and finally dried in vacuum at 60 °C for 12 h. The products prepared with different amounts of KI (0.1 mmol, 0.2 mmol, 0.3 mmol, 0.4 mmol) were denoted as 0.1AgI/Ag_2_WO_4_, 0.2AgI/Ag_2_WO_4_, 0.3AgI/Ag_2_WO_4_, and 0.4AgI/Ag_2_WO_4_, respectively.

### Characterization

The crystalline structure of the samples was studied by using a Bruker D8 Advance X-ray diffractometer (XRD). The images of the morphological structure were observed by a field emission scanning electron microscope (FE-SEM, Hitachi S–4800) and a high-resolution transmission electron microscope (HRTEM, JEOL JEM–2010F). Energy-dispersive X-ray (EDX) spectroscopy coupled with SEM was employed to identify the chemical composition of the sample. UV–vis diffuse reflectance spectra (UV–vis DRS) were recorded on an UV–vis spectrophotometer (Shimadzu, UV-2600).

### Photocatalytic tests

The photocatalytic activity of the as-prepared samples was evaluated by the removal of RhB, MO or 4-CP under visible-light irradiation. A 300 W Xe lamp with a cut-off filter (λ > 400 nm) was used as the light source during the reaction. In each experiment, 10 mg of catalyst was dispersed in RhB (100 mL, 10 mg L^–1^), MO (100 mL, 5 mg L^–1^) or 4-CP (100 mL, 5 mg L^–1^) aqueous solution. Before light illumination, the suspensions were first magnetically stirred in the dark for 1 hour. Then 2 mL of the suspension was collected and the light was switched on. With the light on and under magnetic stirring, 2 mL of the suspension was sampled at given time intervals. All suspensions were centrifuged to remove the catalyst particles. The RhB and MO concentrations were monitored by a UV-2600 spectrometer. The 4-CP concentrations were monitored by high-performance liquid chromatography (HPLC, Agilent 110 series).

The total organic carbon (TOC) experiment was carried out by dispersing 100 mg of 0.3AgI/Ag_2_WO_4_ in RhB (50 mg L^–1^, 100 mL) solution. During the reaction, a 10 mL suspension was sampled every hour and monitored by a TOC analyzer (Shimadzu TOC-VCPH).

Radical trapping experiments were conducted by introducing diverse scavengers (1 mM ammonium oxalate, 1 mM *p*-benzoquinone or 1 mM isopropanol) into the RhB (100 mL, 10 mg L^−1^) solution.

## Supporting Information

File 1 Additional figure.
Degradation of *para*-chlorophenol (4-CP) by 0.3AgI/Ag2WO4 under visible light and the degradation rate constants 4-CP.

## References

[1] Li S, Zhang L, Wang H, Chen Z, Hu J, Xu K, Liu J (2014). Sci Rep.

[2] Jing P, Lan W, Su Q, Xie E (2015). Beilstein J Nanotechnol.

[3] Zhang G, Liu G, Wang L, Irvine J T S (2016). Chem Soc Rev.

[4] Zhang G, Lan Z-A, Wang X (2016). Angew Chem, Int Ed.

[5] Ong W-J, Tan L-L, Ng Y H, Yong S-T, Chai S-P (2016). Chem Rev.

[6] Han W, Li Z, Li Y, Fan X, Zhang F, Zhang G, Peng W (2017). Front Chem (Lausanne, Switz).

[7] Ong W-J (2017). Front Mater.

[8] Kumar S, Kumar A, Bahuguna A, Sharma V, Krishnan V (2017). Beilstein J Nanotechnol.

[9] Huang X, Wang J, Li T, Wang J, Xu M, Yu W, Abed A E, Zhang X (2018). Beilstein J Nanotechnol.

[10] Chen X, Li N, Kong Z, Ong W-J, Zhao X (2018). Mater Horiz.

[11] Zeng S, Kar P, Thakur U K, Shankar K (2018). Nanotechnology.

[12] Wang H, Zhang L, Chen Z, Hu J, Li S, Wang Z, Liu J, Wang X (2014). Chem Soc Rev.

[13] Li S, Shen X, Liu J, Zhang L (2017). Environ Sci: Nano.

[14] Li S, Hu S, Zhang J, Jiang W, Liu J (2017). J Colloid Interface Sci.

[15] Zhang L, Zhang Q, Xie H, Guo J, Lyu H, Li Y, Sun Z, Wang H, Guo Z (2017). Appl Catal, B.

[16] Laatar F, Moussa H, Alem H, Balan L, Girot E, Medjahdi G, Ezzaouia H, Schneider R (2017). Beilstein J Nanotechnol.

[17] Chen C, Ma W, Zhao J (2010). Chem Soc Rev.

[18] Moniz S J A, Shevlin S A, Martin D J, Guo Z-X, Tang J (2015). Energy Environ Sci.

[19] Zhang G, Li G, Lan Z-A, Lin L, Savateev A, Heil T, Zafeiratos S, Wang X, Antonietti M (2017). Angew Chem, Int Ed.

[20] Zhang G, Lan Z-A, Wang X (2017). Chem Sci.

[21] Martin D J, Liu G, Moniz S J A, Bi Y, Beale A M, Ye J, Tang J (2015). Chem Soc Rev.

[22] Wang X, Yang J, Ma S, Zhao D, Dai J, Zhang D (2016). Catal Sci Technol.

[23] Liu B, Li X, Zhao Q, Ke J, Tadé M, Liu S (2016). Appl Catal, B.

[24] Yang S-F, Niu C-G, Huang D-W, Zhang H, Liang C, Zeng G-M (2017). Environ Sci: Nano.

[25] Wang X, Li S, Yu H, Yu J, Liu S (2011). Chem – Eur J.

[26] Xu D, Cheng B, Cao S, Yu J (2015). Appl Catal, B.

[27] Yu C, Li G, Kumar S, Yang K, Jin R (2014). Adv Mater.

[28] Jiao Z, Zhang Y, Yu H, Lu G, Ye J, Bi Y (2013). Chem Commun.

[29] Zhu X, Wang P, Li M, Zhang Q, Rozhkova E A, Qin X, Zhang X, Dai Y, Wang Z, Huang B (2017). Catal Sci Technol.

[30] Wang X, Fu C, Wang P, Yu H, Yu J (2013). Nanotechnology.

[31] Xu D, Cheng B, Zhang J, Wang W, Yu J, Ho W (2015). J Mater Chem A.

[32] Li S, Hu S, Jiang W, Liu Y, Liu J, Wang Z (2017). Mol Catal.

[33] Li J, Yu C, Zheng C, Etogo A, Xie Y, Zhong Y, Hu Y (2015). Mater Res Bull.

[34] Ong W-J, Putri L K, Tan L-L, Chai S-P, Yong S-T (2016). Appl Catal, B.

[35] Tang H, Fu Y, Chang S, Xie S, Tang G (2017). Chin J Catal.

[36] Dutta D P, Singh A, Ballal A, Tyagi A K (2014). Eur J Inorg Chem.

[37] Chen H, Xu Y (2014). Appl Surf Sci.

[38] Pirhashemi M, Habibi-Yangjeh A (2017). J Colloid Interface Sci.

[39] Li Y, Jin R, Fang X, Yang Y, Yang M, Liu X, Xing Y, Song S (2016). J Hazard Mater.

[40] Wang X, Zhan S, Wang Y, Wang P, Yu H, Yu J, Hu C (2014). J Colloid Interface Sci.

[41] Wang X, Li S, Yu H, Yu J (2011). J Mol Catal A: Chem.

[42] Lv J, Dai K, Zhang J, Lu L, Liang C, Geng L, Wang Z, Yuan G, Zhu G (2017). Appl Surf Sci.

[43] Chen J, Li S, Hu S, Jiang W (2017). Mater Lett.

[44] Liu X, Hu J, Li J, Hu Y, Shao Y, Yang H, Tong G, Qian H (2013). Mater Lett.

[45] Pirhashemi M, Habibi-Yangjeh A (2017). Ceram Int.

[46] Li S, Hu S, Jiang W, Liu Y, Liu J, Wang Z (2017). J Colloid Interface Sci.

[47] Li S, Hu S, Xu K, Jiang W, Liu J, Wang Z (2017). Nanomaterials.

[48] Li S, Hu S, Xu K, Jiang W, Liu Y, Leng Z, Liu J (2017). J Colloid Interface Sci.

[49] Zhang J, Ma Z (2017). J Taiwan Inst Chem Eng.

[50] Marschall R (2014). Adv Funct Mater.

[51] Li H, Zhou Y, Tu W, Ye J, Zou Z (2015). Adv Funct Mater.

[52] Shi J (2013). Chem Rev.

