# Supplementary figures and images for: Ag2WO4 nanorods decorated with AgI nanoparticles: Novel and efficient visible-light-driven photocatalysts for the degradation of water pollutants
Source: Beilstein J Nanotechnol. 2018 Apr 27;9:1308–16. doi: 10.3762/bjnano.9.123 (PMC5942385; doi:10.3762/bjnano.9.123)

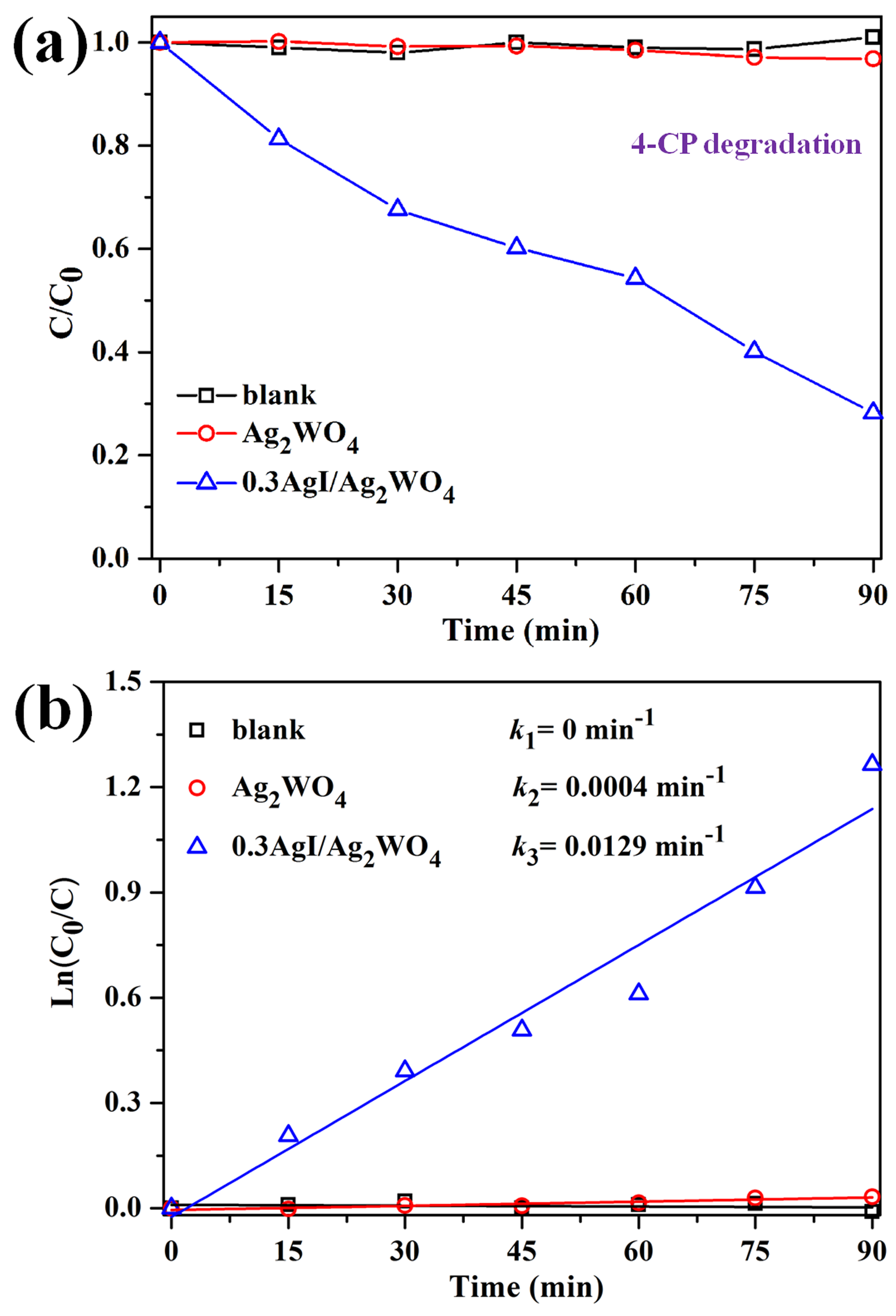

Supplement: File 1 — Additional figure. Degradation of para-chlorophenol (4-CP) by 0.3AgI/Ag2WO4 under visible light and the degradation rate constants 4-CP. [file Beilstein_J_Nanotechnol-09-1308-s001.tif]
